# Complementing Engel Classification: Postoperative Scalp EEG as an Objective Indicator in SEEG‐Guided Radiofrequency Thermocoagulation—A Seizure‐Onset Pattern‐Based Stratified Analysis—

**DOI:** 10.1002/acn3.70553

**Published:** 2026-10-10

**Authors:** Yi‐qing Yang, Rui Zhang, Haiyan Ma, Lu Yang, Xuefeng Qu, Qiqi Chen, Yan Zhang

**Affiliations:** ^1^ Department of Functional Neurosurgery Nanjing Brain Hospital Affiliated to Nanjing Medical University Nanjing China; ^2^ Department of Epilepsy Center Nanjing Brain Hospital Affiliated to Nanjing Medical University Nanjing China

**Keywords:** drug‐resistant epilepsy, low‐voltage fast activity, postoperative electroencephalography, radiofrequency thermocoagulation, seizure‐onset pattern, Stereoelectroencephalography

## Abstract

**Background:**

Stereoelectroencephalography‐guided radiofrequency thermocoagulation (SEEG‐RFTC) is a minimally invasive therapy for drug‐resistant focal epilepsy. However, the relationship between specific seizure‐onset patterns (SOPs), particularly low‐voltage fast activity (LVFA), and postoperative scalp EEG normalization remains inadequately characterized. This study investigates whether LVFA predicts RFTC efficacy and whether postoperative EEG serves as an objective complement to clinical outcome measures.

**Objective:**

To evaluate LVFA‐dominant SOPs as predictors of seizure freedom and scalp EEG normalization after SEEG‐RFTC, and to assess their interaction with MRI findings.

**Methods:**

In a retrospective cohort of 25 patients, seizure‐onset patterns (SOPs) were classified as LVFA‐present (*n* = 13) or LVFA‐absent (*n* = 12). Outcomes included Engel classification and 6‐month postoperative scalp EEG. Seizure freedom and EEG normalization rates were compared, with MRI‐stratified analysis.

**Results:**

The seizure‐free rate was 92.3% (12/13) in the LVFA‐present group versus 33.3% (4/12) in the LVFA‐absent group (*p* = 0.004). EEG normalization was more frequent in LVFA‐present patients (7/8, 87.5%) than in LVFA‐absent patients (4/12, 33.3%; *p* = 0.028); all but one patient with EEG normalization was seizure‐free, the sole exception being an LVFA‐absent patient who remained Engel III. In MRI‐negative patients, LVFA‐present had 100% (6/6) seizure freedom versus 20.0% (1/5) without LVFA (*p* = 0.015). Scalp EEG normalization may serve as a relatively objective marker of complete disintegration of the epileptic network, whereas some patients with persistent EEG abnormalities may still achieve seizure freedom with the aid of antiseizure medications.

**Conclusion:**

In this cohort, LVFA‐present SOPs are strongly associated with favorable RFTC outcomes and may represent a prerequisite for postoperative scalp EEG normalization. Postoperative EEG is a sensitive objective endpoint that complements Engel classification. Systematic PET evaluation in larger cohorts may further refine candidate selection in MRI‐negative patients.

## 1 Introduction

Drug‐resistant focal epilepsy remains a formidable challenge in clinical neuroscience, with approximately one‐third of patients continuing to experience seizures despite adequate trials of antiseizure medications [1, 2]. For these patients, precise localization and complete resection of the epileptogenic zone represent the optimal strategy for achieving long‐term seizure freedom [3, 4]. As the proportion of complex cases—such as those with negative MRI, extensive or multifocal epileptogenic networks, or epileptogenic zones overlapping eloquent cortex—continues to rise, SEEG has progressively replaced subdural grid electrodes as the standard invasive tool for presurgical evaluation [5, 6, 7, 8].

SEEG also enables a minimally invasive therapy: radiofrequency thermocoagulation (RFTC) through the implanted electrodes. By producing focal thermolesions within the presumed SOZ, RFTC converts a diagnostic procedure into a therapeutic intervention without additional surgical trajectories, making it particularly suitable for patients who are not candidates for or refuse open resection [9, 10]. The efficacy of RFTC has been demonstrated across multiple etiologies [11, 12, 13, 14, 15].

The seizure‐onset pattern (SOP) is a fundamental electrophysiological basis for seizure‐onset zone (SOZ) delineation. Multiple classification systems for SOPs have been proposed [16, 17]. In a study of 252 patients, Lagarde et al. characterized eight SOPs and demonstrated that patterns containing low‐voltage fast activity (LVFA) were independently associated with favorable postsurgical outcomes [18, 19]. Pathophysiologically, LVFA reflects inactivation of local inhibitory interneurons and synchronized pyramidal‐cell firing, identifying the epileptogenic core rather than propagation territories [20, 21]. Furthermore, this mechanism can be explained by an imbalance model of inhibitory circuits [22, 23]. In MRI‐negative cases, LVFA frequently corresponds to occult focal cortical dysplasia (FCD), particularly bottom‐of‐sulcus dysplasia, thereby providing a crucial electrophysiological target [24, 25, 26]. However, these associations require further validation.

The Engel classification, however, reliant on subjective seizure reports, cannot ascertain whether the epileptogenic network has been completely disintegrated or capture residual subclinical electrical activity. Postoperative scalp EEG provides an objective, noninvasive, longitudinally trackable measure beyond Engel classification [26]. Postoperative EEG normalization often confirms that the epileptogenic driver has been adequately destroyed [10], but systematic analyses linking postoperative EEG dynamics to preoperative SOP types—especially LVFA—are scarce. Therefore, we performed this retrospective study to include postoperative scalp EEG as an outcome measure alongside Engel classification, and to assess SOPs' prognostic value in a stratified manner.

RFTC offers unique methodological advantages for studying the prognostic value of SOPs. The thermolesions are strictly confined to the electrode contacts that record the seizure onset and early propagation, achieving an intrinsic spatial co‐localization between electrophysiological patterns and clinical responses, and are not influenced by the extent of resection in resective surgery [10, 27]. Therefore, RFTC provides an ideal model to test whether LVFA has independent prognostic significance. This study aimed to: (1) compare postoperative seizure freedom and, importantly, scalp EEG normalization between patients with LVFA‐present and LVFA‐absent SOPs; (2) examine whether EEG normalization represents a functional endpoint that complements Engel classification; and (3) determine whether the LVFA‐EEG correlation is independent of MRI findings.

## 2 Materials and Methods

### 2.1 Patient Selection

Between 2022 and 2024, a total of 43 consecutive patients underwent SEEG implantation at Nanjing Brain Hospital. All 43 patients received RFTC as the initial intervention. Among them, 18 patients subsequently underwent resective surgery because they did not achieve seizure freedom with RFTC alone, after comprehensive evaluation suggested potential benefit and informed consent was obtained. The remaining 25 patients received SEEG‐guided RFTC as the sole therapeutic intervention and did not undergo second‐stage surgery, forming the study cohort, a naturally selected bedside/SEEG‐guided RFTC subgroup. Eligibility required a minimum 12‐month follow‐up. All 25 patients who underwent SEEG‐RFTC during this period were included; no RFTC cases were excluded. Patients undergoing direct resective surgery after SEEG (without RFTC) were not part of this analysis. Data included clinical history, brain MRI [18], F‐FDG PET/CT + MRI fusion, video‐EEG, SEEG recordings, procedural details, 24‐h postoperative scalp EEG at 6 months, and Engel outcome. The ethics committee approved the study (2026‐KY062‐01); all patients provided written informed consent.

### 2.2 Definitions of MRI Status

#### 2.2.1 MRI‐Negative

No definite structural abnormality identified on 3 T epilepsy protocol MRI (3D T1, T2, FLAIR) as independently reviewed by two neuroradiologists with expertise in epilepsy.

#### 2.2.2 MRI‐Positive

Presence of a definite structural lesion (e.g., focal cortical dysplasia, hippocampal sclerosis, periventricular heterotopia, tumor, etc.) on the same protocol.

### 2.3 SEEG Implantation and Presurgical Evaluation

All patients underwent a comprehensive noninvasive workup: prolonged video‐EEG, 3.0 T MRI with epilepsy protocol [18], F‐FDG PET/CT + MRI fusion, magnetoencephalography, and neuropsychological assessment. Each case was discussed in a multidisciplinary conference. SEEG was indicated when the SOZ was suspected to extend beyond the imaging abnormality, involve eloquent cortex, or when MRI was unrevealing. For MRI‐positive patients, SEEG was performed when scalp EEG semiology was discordant with the lesion location, when the lesion was in or near eloquent cortex requiring functional mapping, or when there was suspicion of an extended network beyond the visible lesion.

Depth electrodes (HKHS, Beijing, China) with the following characteristics: 8–18 contacts, 0.8 mm in diameter, 2 mm long, and 1.5 mm inter‐contact distance were implanted under robotic navigation (ROSA, Zimmer Biomet). Post‐implantation CT was fused with volumetric MRI for anatomical localization. Intracranial monitoring lasted 14–28 days; antiseizure medications were reduced to provoke habitual seizures. After capturing sufficient spontaneous seizures, electrical stimulation mapping (50 Hz, 3‐s trains, up to 5.0 mA) was performed.

### 2.4 RFTC Procedure

RFTC was exclusively directed at the epileptogenic zone and the portion of the early propagation zone (EPZ) anatomically contiguous with the SOZ. The SOZ was defined as the cortical region in which ictal discharge patterns first emerged on SEEG. The EPZ was defined as cortical structures engaged during the early phase of seizure propagation with epileptic activity appearing later and with lower intensity relative to the SOZ. The SOZ and EPZ were differentiated based on the temporal sequence and relative intensity of ictal discharges. The SOZ was defined by EEG changes time‐locked to clinical seizure onset, characterized by the earliest appearance and consistent electrographic alterations across multiple spontaneous seizures. In contrast, the EPZ exhibited later onset and lower intensity. EPZ contacts were selected according to the following electrophysiological criteria: (1) a consistent early propagation pattern across multiple spontaneous seizures; (2) confirmation via bipolar montage that discharges occurred after the SOZ but before other regions; and (3) exclusion of contacts demonstrating only late or inconsistent propagation. Radiofrequency energy was delivered using a generator (R2000B, BNS, Beijing, China) with a power‐ramping protocol: starting at 3.0 W, increased in 0.5 W increments every 30–60 s up to 5.5 W, with a maximum temperature cap of 80°C. Antiseizure medications were maintained at pre‐RFTC levels during the procedure.

Post‐ablation SEEG monitoring continued for 24 h to assess acute changes and capture early post‐ablation seizures. Electrodes were then explanted, and a control CT was obtained immediately. At 5 days post‐RFTC, a 3.0 T MRI with T1 and T2/FLAIR sequences was performed to assess lesion characteristics.

### 2.5 Postoperative Scalp EEG and MRI Assessment

Postoperative MRI was performed twice: at 5 days post‐RFTC to evaluate acute lesion volume and edema, and again at 6 months post‐RFTC to assess final lesion volume and chronic gliosis. At the same 6‐month time point, a 24‐h scalp EEG was recorded using a 21‐channel international 10–20 system (referential and bipolar montages), including 30 min of wakefulness, hyperventilation, and photic stimulation. EEGs were independently reviewed by two clinical neurophysiologists blinded to SOP classification and clinical outcomes.

### 2.6 EEG Normalization Definitions

According to routine clinical criteria—namely, based on recordings obtained using the International 10–20 System of Electrode Placement (21 scalp electrodes), interpreted with referential (linked‐ear) and bipolar longitudinal montages, with consideration of the patient's age, state of consciousness, and medication status, and independently reviewed by two clinical neurophysiologists—abnormal EEG was defined as the presence of either of the following: (1) any interictal epileptiform discharges (spikes, sharp waves, spike–wave complexes, or polyspikes); or (2) focal slow waves with morphological abnormality and amplitude ≥ 50% above background activity. Normal EEG was defined as the absence of the above‐mentioned abnormalities.

Postoperative EEG normalization was defined as an EEG meeting the above normal criteria, or showing no interictal epileptiform discharges but with focal slow‐wave activity within the expected postoperative range—a state that may indicate substantial elimination of the primary epileptogenic driver [28]. In this study, all postoperative EEGs that met the above normalization criteria were classified into the normal EEG group.

The above definitions were applied consistently across all postoperative EEG assessments.

### 2.7 Statistical Analysis

Descriptive statistics for continuous variables are reported as mean ± SD (range). For two‐group comparisons, the Mann–Whitney U test was employed to analyze continuous variables that deviated from normality, whereas Fisher's exact test was used to compare categorical variables. A two‐sided significance threshold of *p* < 0.05 was adopted, and all analyses were conducted using SPSS version 26.0.

## 3 Results

### 3.1 Seizure‐Onset Patterns

Based on Lagarde et al. (2019a, 2016), seven categories of ictal electrographic activities were identified: (1) LVFA‐associated patterns (LVFA, baseline shift prior to LVFA, preictal activity prior to LVFA, delta‐brush prior to LVFA, burst of polyspikes prior to LVFA); (2) delta brush‐associated patterns (Delta brush, Delta‐Brush prior to LVFA, Delta brush prior to Beta sharp activity); (3) low‐frequency high‐amplitude periodic spikes (LFPS); (4) sharp activity; (5) beta sharp activity; (6) delta activity; and (7) electrodecrement (Figure 1). Notably, “non‐evolving delta brush”—discussed in subsequent subgroup analyses—is classified under the delta brush‐associated patterns category.

**FIGURE 1:**
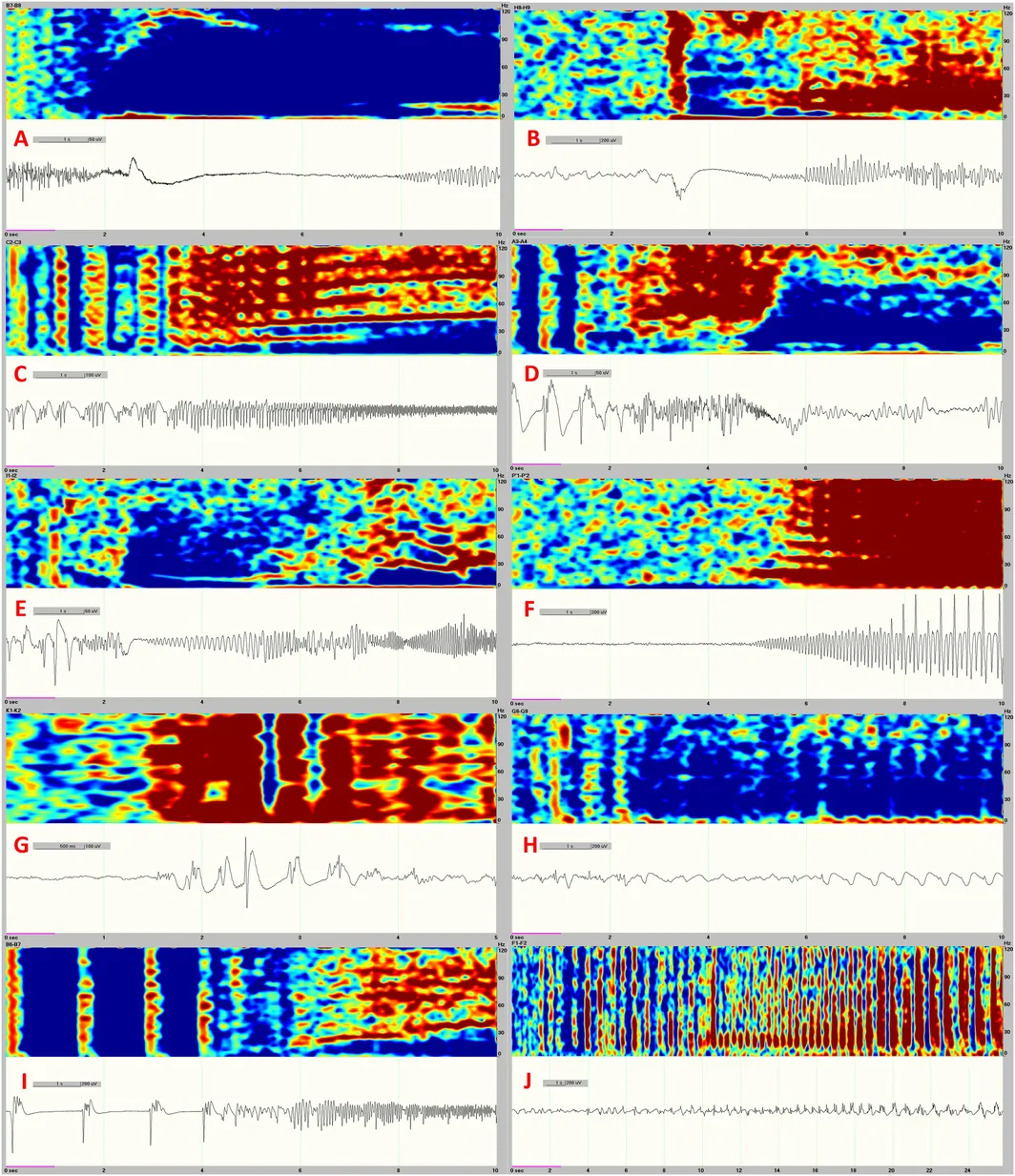
Representative SEEG traces from the majority of the seizure‐onset pattern (SOP) subtypes identified in this study, with the exception of beta sharp activity and electrodecrement. A: LVFA; B: Baseline shift prior to LVFA; C: Preictal activity prior to LVFA; D: Delta‐Brush prior to LVFA; E: Burst of polyspikes prior to LVFA; F: Sharp activity; G: LFPS; H: Delta activity; I: Delta brush prior to beta sharp activity; J: Delta brush. Time and amplitude scales are given after each subfigure label. LVFA, low‐voltage fast activity.

### 3.2 Patient Characteristics and Overall Outcome

The mean number of radiofrequency (RF) electrodes was 2.9 ± 1.1 (range: 2–5). Each bipolar radiofrequency thermocoagulation between two adjacent electrode contacts produced a cortical lesion approximately 7 mm in diameter on 6‐month MRI fusion. The anatomical distribution of ablations is detailed in Table 1. Importantly, there were no statistically significant differences between the LVFA‐present and LVFA‐absent groups with respect to the number of RF electrodes (Mann–Whitney U = 72.0, *p* = 0.646) or the total number of RF contacts (*U* = 73.5, *p* = 0.719). Mean follow‐up was 31.6 months (range 16.1–44.4). The overall Engel class I rate was 64.0% (16/25). Individual patient data are provided in Table 2.

**TABLE 1:** Individual clinical data.

Engel	Pt	MRI	EEG	SOP	Target	Elec. (*n*)	Cont. (*n*)	T
I	1	Neg.	Nor.	Baseline shift prior to LVFA	R. OrG; R. Ant. ReG.	2	8 + 5	13
I	5	FCDII	Nor.	Delta‐Brush prior to LVFA	L. Ant.‐Mid. CG; L. Ant. SFG.	5	4 + 6 + 6 + 5 + 6	27
I	6	FCDII	Nor.	Preictal activity prior to LVFA	L. SFS (Ant. to PreCS).	5	5 + 5 + 4 + 5 + 5	24
I	7	PNH	Nor.	LVFA	R. OL	2	6 + 5	11
I	8	TSC	Ab.	Sharp activity	R. Mid. PreCG	2	7 + 7	14
I	9	Neg.	Nor.	Baseline shift prior to LVFA	R. SFG; R. Post. SFS.	3	8 + 4 + 4	16
I	10	Neg.	No	LVFA	R. IFG (Op & Tri)	2	6 + 6	12
I	12	Neg.	Nor.	LVFA	R. AIns	2	4 + 2	6
I	14	Ence.	Ab.	LFPS	R. Inf. PoCG; R. IPL.	4	6 + 5 + 5 + 3	19
I	15	HS	Ab.	Burst of polyspikes prior to LVFA	R. Ant.‐Mid. TL; R. Hipp. & Amyg.	4	4 + 4 + 3 + 2	13
I	16	Neg.	Ab.	LVFA	R. PL; R. SMG.	5	5 + 6 + 6 + 5 + 4	26
I	17	Neg.	Nor.	Burst of polyspikes prior to LVFA	pACC	2	4 + 3	7
I	19	HS	No	LVFA	R. Hipp. & Amyg.	2	4 + 4	8
I	20	gliosis	Ab.	Sharp activity	L. OL; L. PCun.	3	7 + 6 + 5	18
I	22	Ence.	Ab.	Delta brush	L. PL; L. PCun.	2	5 + 7	12
I	23	Neg.	No	Sharp activity	L. Hipp. & Amyg.	2	4 + 4	8
II	4	Neg.	Ab.	Beta sharp activity	L. ACC; L. Ant. IFG.	2	8 + 6	14
III	2	Ence.	Ab.	Baseline shift prior to LVFA	R. OFC; R. ReG.	2	6 + 8	14
III	3	Neg.	Ab.	Electrodecrement	R. SPL; R. Mid. PoCG.	4	6 + 6 + 4 + 3	19
III	21	HS	Ab.	Beta sharp activity	L. PHG; L. Amyg.	2	4 + 4	8
III	25	FCDII	Nor.	Delta brush	L. spACC	3	4 + 4 + 4	12
IV	11	FCDII	No	Delta brush prior to Beta sharp activity	R. Mid. PoCG	4	9 + 9 + 6 + 5	29
IV	13	Ence.	Ab.	Delta activity	R. PCun.	3	3 + 3 + 4	10
IV	18	Neg.	Ab.	Delta activity	R. MCC	3	3 + 7 + 6	16
IV	24	Neg.	No	Delta activity	R. Hipp. & Amyg.	3	3 + 3 + 3	9

*Note:* Anatomical abbreviations are provided in Table S1.

Abbreviations: Ab., abnormal post‐RFTC EEG; Cont., number of RF contacts; EEG, post‐RFTC electroencephalogram; Elec., number of RF electrodes; Ence., encephalomalacia; Engel, Engel classification of seizure outcome; FCDII, focal cortical dysplasia type II; HS, hippocampal sclerosis; LFPS, low‐frequency high‐amplitude periodic spikes; LVFA, low‐voltage fast activity; MRI, preoperative magnetic resonance imaging finding; Neg., negative (no lesion detected on MRI); No, post‐RFTC EEG not performed; Nor., normal post‐RFTC EEG; PNH, periventricular nodular heterotopia; Pt, patient number; SOP, seizure‐onset pattern; T, total number of RF contacts; TSC, tuberous sclerosis complex.

**TABLE 2:** Patient features.

	RFTC
Patients	25
Sex (F/M)	12/13
Age at epilepsy onset (years, range)	13.7 (0–28)
Epilepsy duration (years, range)	15.7 (4–30)
Age at SEEG (years, range)	29.9 (12–54)
Electrodes (trajectories; *n*, sum, SD)	9.84 (246, 2.48)
Contacts per electrode (range)	8–18
Seizures (*n*, sum, SD)	4.48 (112, 2.5)
RFTC outcome	
Seizure free	16
Non free (II, III, IV)	9 (1, 4, 4)
Follow‐up duration (months, range)	31.6 (16.1–44.4)

*Note:* For quantitative data, we reported the mean value, sum, and the range.

Abbreviations: F, female; M, male; RFTC, radiofrequency thermocoagulation; y, year.

### 3.3 Postoperative Scalp EEG as an Outcome Measure

Twenty patients underwent postoperative EEG: 8 normal, 12 abnormal, 5 not performed (due to patient refusal or loss to follow‐up; these patients were excluded from EEG‐based analyses).

### 3.4 Relationship Between Postoperative EEG and Seizure Freedom

Seizure‐free rates were 87.5% (7/8) with normal EEG, 50.0% (6/12) with abnormal EEG, and 60.0% (3/5) without EEG (Table 3). The difference between normal and abnormal EEG groups was not statistically significant (*p* = 0.158). Normal EEG had a positive predictive value of 87.5% for seizure freedom in this cohort.

**TABLE 3:** Relationship between postoperative EEG and outcome.

Postoperative EEG	Total *n*	Engel I	Engel II–IV	Seizure‐free (%)	*p*
Normal	8	7	1	87.5	0.158
Abnormal	12	6	6	50.0	—
Not performed	5	3	2	60.0	—

*Note:* *p* value was obtained using Fisher's exact test for the comparison of seizure‐free rates between the normal and abnormal postoperative EEG groups.

### 3.5 Subgroup Analysis of Patients With Abnormal EEG

Among 12 patients with abnormal EEG, seizure‐free rates were 75.0% (3/4) in the LVFA‐present group and 37.5% (3/8) in the LVFA‐absent group (*p* = 0.546; Table 4), indicating that persistent EEG abnormalities do not uniformly portend poor prognosis when clinical seizures are controlled.

**TABLE 4:** Stratified analysis of patients with abnormal postoperative EEG by SOP.

SOP group	Total *n*	Engel I	Engel II–IV	Seizure‐free (%)	*p*
LVFA‐present	4	3	1	75.0	0.546
LVFA‐absent	8	3	5	37.5	—

*Note:* *p* value was obtained using Fisher's exact test.

### 3.6 SOP Composition in Normal Versus Abnormal EEG

LVFA‐present SOPs were significantly more frequent in patients with normal EEG (7/8, 87.5%) than in those with abnormal EEG (4/12, 33.3%; *p* = 0.028; Table 5). One LVFA‐absent patient (No. 25, delta brush onset) achieved EEG normalization without seizure freedom (Engel III), underscoring that scalp EEG and Engel classification capture complementary dimensions of therapeutic success. In the majority of patients, the cascade of LVFA onset → precise lesioning → network disintegration → EEG normalization was supported.

**TABLE 5:** Comparison of SOP composition between patients with normal and abnormal postoperative EEG.

SOP group	Normal EEG (*n* = 8)	Abnormal EEG (*n* = 12)	*p*
LVFA‐present	7 (87.5%)	4 (33.3%)	0.028
LVFA‐absent	1 (12.5%)	8 (66.7%)	—

*Note:* *p* value was obtained using Fisher's exact test.

### 3.7 Stratified Analysis by SOP and MRI

#### 3.7.1 Impact of SOP on Prognosis

The seizure‐free rate was 92.3% (12/13) in the LVFA‐present group versus 33.3% (4/12) in the LVFA‐absent group (*p* = 0.004; Table 6). In the LVFA‐absent group, the 4 patients who achieved seizure freedom included 3 with sharp activity and 1 with non‐evolving delta brush.

**TABLE 6:** Overall outcome and subgroup analysis based on MRI and LVFA.

Group	Total *n*	Engel I	Engel II–IV	Seizure‐free (%)	*p*
Overall	25	16	9	64.0	—
MRI‐positive	14	9	5	64.3	1.000
MRI‐negative	11	7	4	63.6	—
LVFA‐present	13	12	1	92.3	0.004
LVFA‐absent	12	4	8	33.3	—

*Note:* *p* values were obtained using Fisher's exact test. MRI‐positive versus MRI‐negative, *p* = 1.000; LVFA‐present versus LVFA‐absent, *p* = 0.004.

#### 3.7.2 Interaction Between MRI and SOP

Stratified analysis (Table 7) showed: in MRI‐positive patients, LVFA‐present 85.7% (6/7) seizure‐free versus LVFA‐absent 42.9% (3/7) (*p* = 0.266); in MRI‐negative patients, LVFA‐present 100% (6/6) versus LVFA‐absent 20.0% (1/5) (*p* = 0.015). Thus, in this cohort, LVFA status demonstrated a stronger association with RFTC efficacy than MRI positivity alone.

**TABLE 7:** Stratified interaction analysis of MRI findings and LVFA.

MRI finding	LVFA status	*n*	Engel I	Engel II–IV	Seizure‐free (%)	*p*
MRI‐positive	LVFA‐present	7	6	1	85.7	0.266
MRI‐positive	LVFA‐absent	7	3	4	42.9	—
MRI‐negative	LVFA‐present	6	6	0	100	0.015
MRI‐negative	LVFA‐absent	5	1	4	20.0	—

*Note:* *p* values were obtained using Fisher's exact test for the comparison between LVFA‐present and LVFA‐absent within the same MRI status.

#### 3.7.3 SOP Composition in Engel Class Outcomes

In the Engel I group (*n* = 16), 75.0% had LVFA‐present patterns versus 11.1% (1/9) in non‐Engel I patients (*p* = 0.003; Table 8). Among Engel I patients with normal EEG (*n* = 7), 100% had LVFA‐present SOPs, whereas those with abnormal EEG (*n* = 6) showed 50% LVFA‐present, 50% LVFA‐absent (*p* = 0.070). EEG normality further stratifies network disruption within clinically seizure‐free patients.

**TABLE 8:** Comparison of SOP composition between Engel I and non‐Engel I patients, with stratification of Engel I patients by postoperative EEG status.

SOP group	Engel I, normal EEG (*n* = 7)	Engel I, abnormal EEG (*n* = 6)	Engel I, no EEG (*n* = 3)	Engel I total (*n* = 16)	Non‐Engel I (*n* = 9)	Total (*n* = 25)
LVFA‐present	7 (100%)	3 (50.0%)	2 (66.7%)	12 (75.0%)	1 (11.1%)	13
LVFA‐absent	0 (0%)	3 (50.0%)	1 (33.3%)	4 (25.0%)	8 (88.9%)	12

*Note:* Values are *n* (%). The Engel I group comprised 16 patients, stratified by postoperative EEG status: 7 with normal EEG, 6 with abnormal EEG, and 3 without postoperative EEG. The Engel I total column represents the sum of the three EEG‐stratified Engel I columns. Comparison of SOP composition between the Engel I group (LVFA‐present 12/16, 75.0%) and the non‐Engel I group (1/9, 11.1%): Fisher's exact test, *p* = 0.003. Comparison between Engel I with normal EEG and Engel I with abnormal EEG groups: Fisher's exact test, *p* = 0.070.

#### 3.7.4 Prognosis of Specific SOP Subtypes

Table 9 shows that all LVFA‐present subtypes achieved high seizure‐free rates (100% for LVFA, burst of polyspikes prior to LVFA, preictal activity prior to LVFA, delta‐brush prior to LVFA, and LFPS; 66.7% for baseline shift prior to LVFA). Among LVFA‐absent subtypes, sharp activity had 100% seizure freedom, non‐evolving delta brush 50%, while beta sharp activity, delta activity, electrodecrement, and delta brush prior to beta sharp activity all had 0% seizure freedom.

**TABLE 9:** Prognostic distribution of specific SOP subtypes.

SOP subtype	Total *n*	Engel I	Engel II	Engel III	Engel IV	Seizure‐free (%)
LVFA	5	5	0	0	0	100
Baseline shift prior to LVFA	3	2	0	1	0	66.7
Burst of polyspikes prior to LVFA	2	2	0	0	0	100
Preictal activity prior to LVFA	1	1	0	0	0	100
Delta‐Brush prior to LVFA	1	1	0	0	0	100
LFPS	1	1	0	0	0	100
Sharp activity	3	3	0	0	0	100
Delta brush	2	1	0	1	0	50
Beta sharp activity	2	0	1	1	0	0
Delta activity	3	0	0	0	3	0
Electrodecrement	1	0	0	1	0	0
Delta brush prior to Beta sharp activity	1	0	0	0	1	0

## 4 Discussion

Although the overall seizure‐free rate of SEEG‐guided radiofrequency thermocoagulation (SEEG‐RFTC) is generally lower than that of conventional resective surgery, a considerable proportion of patients may still benefit from this minimally invasive approach [11, 15]. The success of this treatment depends largely on accurate identification of the core epileptogenic zone through SEEG and subsequent validation via targeted thermocoagulation [10, 29].

During the study period, 43 consecutive patients at our institution all received RFTC as the initial intervention. Of these, 18 subsequently underwent resective surgery because they did not achieve seizure freedom with RFTC alone, after comprehensive evaluation suggested potential benefit and informed consent was obtained. Our strategy was based on the findings of Bourdillon et al. [10] and Dannhoff et al. [30], who reported that even transient post‐coagulation improvement carries a high positive predictive value for subsequent surgical success. The remaining 25 patients did not undergo second‐stage surgery, forming the study cohort, a naturally selected bedside/SEEG‐guided RFTC subgroup. The nine patients who did not achieve seizure freedom within this cohort did not proceed to second‐stage surgery: five patients were satisfied with a marked reduction in seizure burden, and four patients declined craniotomy after shared decision‐making. The overall seizure‐free rate for all 43 patients was 37.2% (16/43), consistent with the 23%–50% reported in the literature [11, 15].

RFTC offers a unique advantage over resective surgery in that thermolesions are strictly confined to the electrode contacts that recorded the ictal onset, achieving an intrinsic spatial co‐localization between the recorded seizure‐onset pattern (SOP) and the clinical response [28, 31], and eliminating confounding factors such as the extent of resection and adequacy of margins [10]. Therefore, SEEG‐RFTC constitutes an ideal model to test whether specific SOPs, such as low‐voltage fast activity (LVFA), carry independent prognostic significance beyond their association with the underlying structural pathology.

In this cohort of 25 patients who received RFTC alone, LVFA‐present SOPs were associated with significantly more favorable RFTC outcomes (92.3% seizure‐free vs. 33.3% in the LVFA‐absent group, *p* = 0.004), and postoperative scalp EEG normalization at 6 months was significantly more frequent in the LVFA‐present group (87.5% vs. 33.3%). These observations suggest a potential prognostic cascade: LVFA may guide target selection, RFTC lesioning may disrupt the driving core, and this functional effect may be reflected as EEG normalization. However, these findings are preliminary and require confirmation in larger, prospective cohorts.

### 4.1 The Pathophysiological Rationale for LVFA as a Targeting Biomarker

LVFA is considered one of the most reliable electrophysiological markers of the core epileptogenic network. It arises from local interneuron inactivation and synchronized pyramidal firing [21], a mechanism supported by the inhibitory circuit imbalance model, in which impaired GABAergic inhibition leads to disinhibition of excitatory pyramidal cells and subsequent high‐frequency rhythmic discharges [22, 23, 32]. Shiri et al. further demonstrated, using optogenetic control of parvalbumin‐positive interneurons, the direct involvement of interneuronal networks in LVFA initiation [21]. By contrast, slow‐wave or electrodecremental patterns typically represent propagation rather than the core epileptogenic zone [17, 19]. Our findings are consistent with this framework: in the 25‐patient RFTC‐only cohort, the seizure‐free rate was 92.3% in the LVFA‐present group versus 33.3% in the LVFA‐absent group (*p* = 0.004), suggesting that ablation targeting LVFA‐onset regions is more likely to directly disrupt the epileptogenic driver.

Lagarde et al. [19] demonstrated in 252 consecutive patients that LVFA is associated with favorable outcomes, and Abdallah et al. [20] confirmed a significant association between LVFA and seizure‐free outcome in their systematic review. Our study extends these findings to the RFTC setting. In a pure bedside/SEEG‐guided RFTC cohort free from confounding by subsequent resection—in contrast to patients who proceeded to open surgery (craniotomy) after RFTC—the prognostic distinction of LVFA was particularly pronounced. Intraoperative RFTC performed under general anesthesia represents a distinct application of this technique [33, 34, 35]. Moreover, among MRI‐negative patients who received RFTC alone, the LVFA‐present group achieved 100% seizure freedom, suggesting that this electrophysiological marker may have special targeting value in patients with occult focal cortical dysplasia (FCD), particularly bottom‐of‐sulcus dysplasia [25, 26, 36, 37].

### 4.2 Postoperative Scalp EEG as an Objective Complement to Engel Classification

The Engel classification, despite its widespread use, has inherent limitations. It relies on patient‐reported seizure counts and subjective recall, which may be inaccurate, especially in patients with subtle or nocturnal seizures. Furthermore, Engel I does not necessarily guarantee that the epileptogenic network has been completely disrupted; residual subclinical electrical activity may persist and later evolve into clinical seizures, particularly after medication tapering. This uncertainty complicates clinical decisions regarding antiseizure medication withdrawal and long‐term prognosis.

Postoperative scalp EEG offers a noninvasive, objective, and longitudinally trackable measure that directly reflects cortical electrical activity. EEG normalization—defined as the complete absence of interictal epileptiform discharges without focal slowing beyond the expected postoperative range—may indicate that the primary epileptogenic driver has been substantially eliminated [28]. In our series, 7 of 8 patients (87.5%) with normal postoperative EEG were seizure‐free, and 7 of the 8 belonged to the LVFA‐present group. The single exception, a patient with delta brush onset who achieved EEG normalization but remained Engel III, underscores that scalp EEG and Engel classification capture complementary dimensions of therapeutic success. All patients maintained their pre‐RFTC antiseizure medication regimens unchanged at the 6‐month follow‐up. In contrast, among seizure‐free patients with abnormal EEG, the LVFA‐present rate was only 50%, suggesting incomplete network disruption despite clinical remission. This dissociation raises the possibility that Engel I with a normal EEG may represent a more complete therapeutic outcome than Engel I with persistent EEG abnormalities and might guide safer medication withdrawal.

Several studies have highlighted the potential prognostic value of postoperative EEG in epilepsy surgery. Bourdillon et al. [28] noted that post‐RFTC EEG changes can reflect the extent of lesioning and may help predict long‐term seizure control. Luo et al. [26] recently demonstrated that postoperative EEG normalization correlates with favorable outcomes in MRI‐negative patients. Gula et al. [38] showed that local RFTC lesions can affect distant brain connectivity and excitability, providing insights into how network changes following RFTC may relate to seizure outcomes. Bolzan et al. [39] explored the correspondence between scalp EEG and SEEG seizure‐onset patterns, further supporting the utility of scalp EEG as a noninvasive surrogate. Our data extend these findings by suggesting a possible link between postoperative EEG status and preoperative SOP type. The most novel feature of this work is that by targeting LVFA with SEEG‐guided RFTC, one can be highly confident that postoperative scalp EEG normalization will occur, a functional outcome not captured by seizure freedom alone.

### 4.3 MRI and SOP Interaction: LVFA Association May be Independent of MRI Findings

In this cohort, MRI positivity did not significantly predict outcome (*p* = 1.000). In MRI‐negative patients, the LVFA‐present group achieved 100% seizure freedom, compared with 20.0% in the LVFA‐absent group (*p* = 0.015). Even in MRI‐positive patients, those with LVFA had notably higher rates (85.7% vs. 42.9%). These findings suggest that, in this selected cohort, the electrophysiological signature, particularly LVFA, may be more closely associated with RFTC efficacy than structural MRI findings alone. However, this observation should be interpreted cautiously, as the number of MRI‐negative patients was small (*n* = 11), and the absence of histopathological confirmation limits definitive conclusions.

Notably, all MRI‐negative patients with LVFA‐present SOPs in this cohort achieved seizure freedom (Figure 2). If such patients also exhibit PET hypometabolism concordant with the SEEG‐defined onset zone, they may represent an “electro‐metabolic” double‐positive subgroup with favorable outcomes, potentially reflecting underlying occult bottom‐of‐sulcus focal cortical dysplasia [25, 26, 40]. SEEG has an irreplaceable role in the presurgical evaluation of MRI‐negative epilepsy [41, 42, 43], and recent work by Luo et al. [26] has further characterized the electrophysiological and histopathological features of this entity.

**FIGURE 2:**
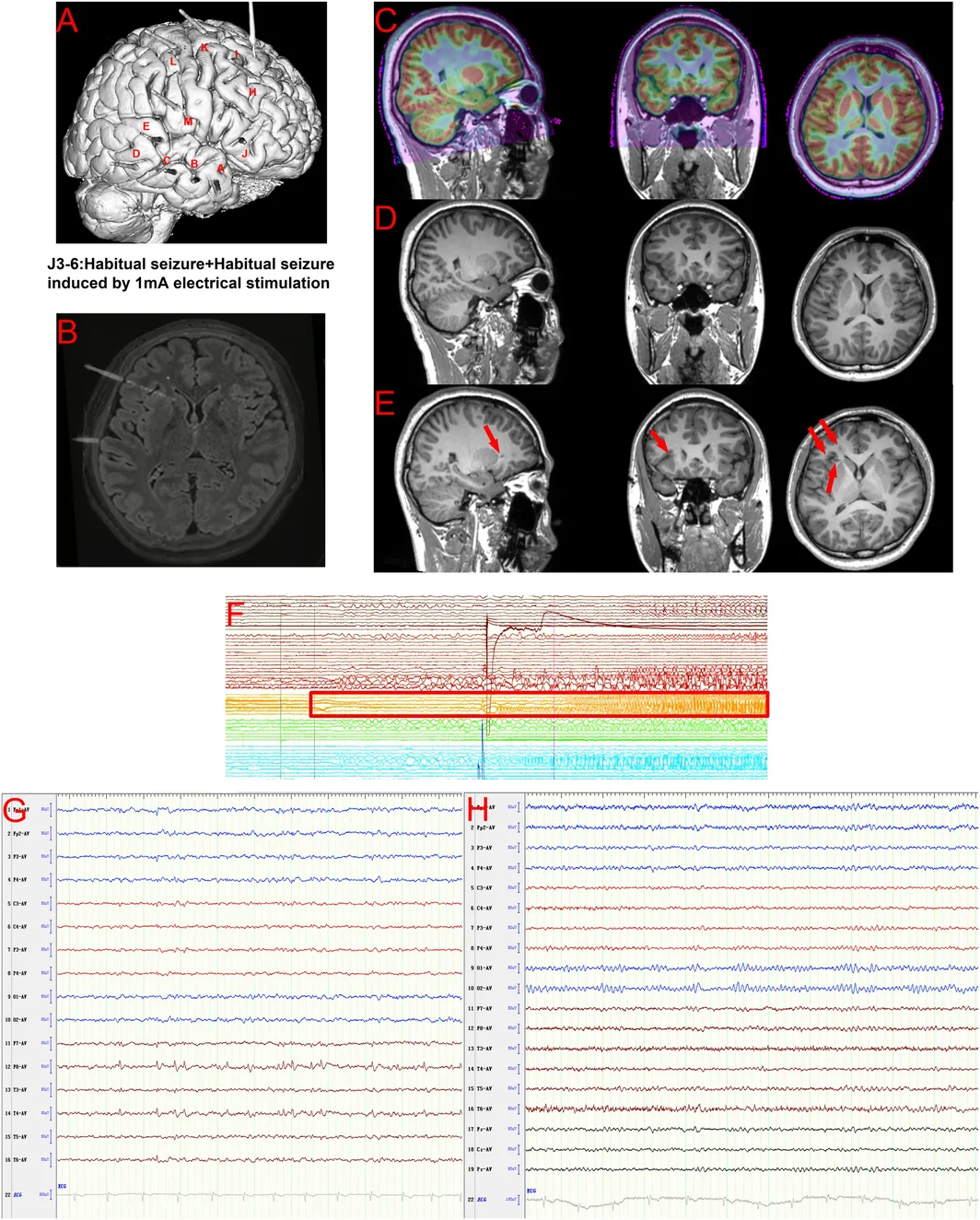
No. 12: An MRI‐negative patient with LVFA onset who achieved seizure freedom and EEG normalization after SEEG‐guided RFTC. A: 3D reconstruction of the brain surface was performed using coregistered T2‐FLAIR and CT images to display the actual positions of the intracranial depth electrodes; B: The fused T2‐FLAIR and CT images clearly demonstrate the actual placement of electrode J, allowing visualization of each individual contact. C: Preoperative MRI‐PET; D: Preoperative MRI T1; E: MRI T1 at 6 months post‐RFTC (arrows indicate the postoperative thermocoagulation changes). F: SEEG identified LVFA onset at contacts J3–J6 (marked by the red box), and the ictal sequence was as follows: Impaired awareness → bilateral manual automatisms → tonic neck posturing → forced head version toward the left and upward. G: Preoperative scalp EEG showed focal periodic epileptiform discharges at F8 and T4. H: At 6 months postoperatively, EEG revealed no preoperative rhythmic discharges and was reported normal by two independent doctors. Panel H displays EEG epochs contemporaneous with Panel G. The patient became seizure‐free after RFTC, and there were no neurological deficits during a follow‐up of more than 1 year.

### 4.4 Observations Regarding Non‐LVFA Patterns

In this cohort, patients with delta activity, beta sharp activity, or electrodecrement had 0% seizure freedom, which may suggest that these patterns indicate propagation zones rather than the core epileptogenic zone. Even in MRI‐positive cases without LVFA, the seizure‐free rate was only 42.9%, and none achieved EEG normalization. These observations raise the concern that RFTC in such situations may risk leaving the true epileptogenic core intact.

However, sharp activity achieved 100% seizure freedom (3/3) and non‐evolving delta brush 50% (1/2) in our small sample, suggesting that a minority of patterns without fast activity might still originate from the epileptogenic focus. This is consistent with previous reports where certain sharp‐wave patterns appeared amenable to intervention [44]. For specific etiologies such as periventricular nodular heterotopia, RFTC outcomes have also been reported to be suboptimal when LVFA is absent [45, 46]. Importantly, exceptions such as sharp activity still lacked EEG normalization in our series, which may indicate a potentially less durable therapeutic mechanism. This observation supports the hypothesis that non‐LVFA onset patterns may reflect more diffuse network phenomena, where focal ablation can still eliminate clinical seizures but may not completely silence the network [17].

### 4.5 Contextualizing the Seizure‐Free Rate

The overall seizure‐free rate of 37.2% (16/43) among all 43 patients aligns with the 23%–50% reported in previous meta‐analyses [11, 15], confirming that our patient population and treatment protocol are broadly representative of published series. The 64% Engel I rate observed in the 25‐patient bedside/SEEG‐guided RFTC cohort is notably higher, but this difference requires careful interpretation. Since all 43 patients initially received RFTC, and the 18 who proceeded to resection did so specifically because RFTC failed to control their seizures, the RFTC‐only cohort was naturally selected. Thus, the 64% figure should not be interpreted as the expected efficacy in unselected patients, but rather as the overall seizure‐free rate within this selected cohort—serving as a reference for the between‐group comparisons (LVFA‐present vs. LVFA‐absent) that constitute the core of our analysis.

This study design, however, does not undermine our primary analysis—rather, it provides a valuable framework. By focusing on the 25 patients who received RFTC alone, we were able to examine the relationship between SOP type and RFTC outcomes under homogeneous conditions, free from the confounding effects of subsequent resective procedures. The marked difference between LVFA‐present and LVFA‐absent groups (92.3% vs. 33.3%) suggests that SOP type, particularly LVFA, may serve as an important predictor of RFTC responsiveness. This finding is consistent with Lagarde et al. [19] and Abdallah et al. [20], who reported that LVFA is associated with favorable surgical outcomes.

### 4.6 A Tentative Clinical Decision Framework for RFTC as a Final Treatment

Based on the observations from this study, we propose a preliminary clinical decision‐making framework to be considered both before RFTC and during post‐treatment assessment.

For treatment decision‐making, our data indicate that SOP type is closely associated with RFTC efficacy. When SEEG reveals an LVFA‐characterized SOP, RFTC may be considered as a potentially definitive treatment option regardless of MRI findings; if the patient is also MRI‐negative with PET hypometabolism concordant with the epileptogenic zone, the prognosis may be even more favorable. Both scenarios are more likely to achieve seizure freedom after RFTC. Conversely, when the SOP is characterized by slow‐wave, electrodecremental, evolving sharp, sharp activity, or non‐evolving delta brush patterns—non‐LVFA patterns—sustained benefit from RFTC appears limited, and the value of the procedure may be more diagnostic in nature. Some patients may experience transient improvement, but the probability of completely eliminating the epileptogenic network is low. Therefore, clinical decision‐making for such patients should be more cautious, with multimodal assessment when necessary, and early evaluation for second‐stage resective surgery should be considered.

Additionally, LVFA belongs to the broad family of fast ictal rhythms that overlap conceptually with high‐frequency oscillations (HFOs, 80–500 Hz), which are increasingly recognized as biomarkers of the epileptogenic network; bridging these perspectives may help unify SEEG‐based targeting strategies across different schools of thought. Even in patients without LVFA, SEEG‐guided RFTC may still be useful by reducing the number of trajectories required in subsequent resective procedures (e.g., thermocoagulation of the amygdala followed by intraoperative hippocampal ablation in mesial temporal lobe epilepsy).

For outcome assessment, postoperative scalp EEG can serve as an important objective complement to Engel classification. In this cohort, EEG normalization was significantly more frequent in LVFA‐positive patients (7/8, 87.5%) than in LVFA‐absent patients (4/12, 33.3%); however, some Engel I patients still exhibited persistent interictal discharges on postoperative EEG, and one LVFA‐absent patient achieved EEG normalization without seizure freedom, suggesting that the epileptogenic network may not have been completely disintegrated. Therefore, EEG status not only helps confirm whether ablation has been adequate, but may also provide a reference for postoperative antiseizure medication tapering.

It should be emphasized that this framework is based on a single‐center retrospective study with a limited sample size; the conclusions are preliminary and require prospective validation before clinical application.

## 5 Limitations

Our findings should be interpreted in the context of several limitations. First, the sample size (*n* = 25) is small and limits the statistical power of subgroup analyses. Second, the retrospective design and the absence of postoperative EEG in 5 patients may introduce selection bias. Third, without histopathological confirmation [47], the diagnosis of FCD in MRI‐negative cases remains electrophysiologically inferred. Fourth, the absence of a control group limits causal inference. Fifth, as highlighted by Abdallah et al. [20], SOP terminology varies considerably across studies, which complicates cross‐study comparisons. Sixth, this cohort was naturally selected, and the higher seizure‐free rate in this cohort likely reflects selection bias rather than a universal treatment effect. Finally, the short follow‐up duration (mean 31.6 months) does not allow assessment of long‐term durability. Larger prospective studies with systematic EEG follow‐up, histopathological correlation, and longer observation periods are needed to validate these preliminary findings.

## 6 Conclusion

In this cohort of 25 patients who received RFTC alone, the LVFA‐present group had significantly better RFTC outcomes than the LVFA‐absent group (92.3% seizure‐free vs. 33.3%, *p* = 0.004). Postoperative scalp EEG normalization was significantly more frequent in patients with LVFA‐present SOPs (7/8, 87.5% vs. 4/12, 33.3%), and nearly all patients with EEG normalization were seizure‐free. One LVFA‐absent patient achieved EEG normalization without seizure freedom. These findings suggest that RFTC targeting the LVFA onset region may be associated with both superior seizure control and scalp EEG normalization, and that postoperative EEG may serve as a useful objective complement to Engel classification, helping to assess whether the epileptogenic driver has been adequately disrupted and potentially guiding decisions about antiseizure medication withdrawal. However, these conclusions are preliminary and require validation in larger, prospective, multicenter studies.

In MRI‐negative patients within this cohort, if SEEG corroborates LVFA onset, favorable outcomes may be achievable, possibly reflecting underlying occult bottom‐of‐sulcus FCD; PET concordance may further refine patient selection. Such patients should not be excluded from RFTC solely on the basis of MRI negativity, but decisions should be made on a case‐by‐case basis. For slow‐wave, electrodecremental, or sharp‐wave onset patterns lacking LVFA—with the exception of certain subtypes—RFTC may frequently fail to encompass the authentic epileptogenic focus, and the risk of persistently abnormal postoperative EEG appears high; such patients may be better evaluated for second‐stage resective surgery.

## Author Contributions

Y.‐q.Y. conceived and designed the study, performed the statistical analysis, and drafted the manuscript. R.Z. performed the SEEG‐guided radiofrequency thermocoagulation procedures and contributed to clinical data acquisition. H.M. and L.Y. acquired the clinical and electrophysiological data and contributed to the review and classification of postoperative scalp EEG. X.Q. contributed to clinical data acquisition and patient follow‐up. Q.C. contributed to SEEG data collection and to the classification of seizure‐onset patterns. Y.Z. contributed to data curation, statistical analysis, and the preparation of tables and figures. All authors critically revised the manuscript for important intellectual content, approved the final version to be published, and agree to be accountable for all aspects of the work.

## Funding

The authors have nothing to report.

## Conflicts of Interest

The authors declare no conflicts of interest.

## Supporting information


**Figure S1:** Postoperative MRI Images of Some Patients. Postoperative MRI images of patients who underwent SEEG‐guided radiofrequency thermocoagulation. The patient number and radiofrequency target(s) are indicated above each panel.

**Table S1:** Anatomical Abbreviations. Abbreviations and full terms of the anatomical structures used to describe the radiofrequency thermocoagulation targets in this study.

## Data Availability

The data that support the findings of this study are available from the corresponding author upon reasonable request.
