# Supplementary material for: Complementing Engel Classification: Postoperative Scalp EEG as an Objective Indicator in SEEG‐Guided Radiofrequency Thermocoagulation—A Seizure‐Onset Pattern‐Based Stratified Analysis—
Source: Ann Clin Transl Neurol. 2026 Oct 10:10.1002/acn3.70553. Online ahead of print. doi: 10.1002/acn3.70553 (PMC13653232; doi:10.1002/acn3.70553)
Supplement: Supplementary file 1 [file ACN3-9999-0-s001.docx]

**Supplementary Table 1. Anatomical Abbreviations**

| Abbreviation | Full terms |
| --- | --- |
| ACC | Anterior Cingulate Cortex |
| AIns | Anterior Insula |
| Amyg. | Amygdala |
| Ant. IFG | Anterior Inferior Frontal Gyrus |
| Ant. ReG | Anterior Rectal Gyrus |
| Ant. SFG | Anterior Superior Frontal Gyrus |
| Ant.-Mid. CG | Anterior-Middle Cingulate Gyrus |
| Ant.-Mid. TL | Anterior-Middle Temporal Lobe |
| Hipp. & Amyg. | Hippocampus and Amygdala |
| IFG (Op & Tri) | Inferior Frontal Gyrus (pars Opercularis & Triangularis) |
| Inf. PoCG | Inferior Postcentral Gyrus |
| IPL | Inferior Parietal Lobule |
| MCC | Midcingulate Cortex / Middle Cingulate Cortex |
| Mid. PoCG | Middle Postcentral Gyrus |
| Mid. PreCG | Middle Precentral Gyrus |
| OFC | Orbitofrontal Cortex |
| OL | Occipital Lobe |
| OrG | Orbital Gyrus |
| pACC | pregenual Anterior Cingulate Cortex |
| PCun. | Precuneus |
| PHG | Parahippocampal Gyrus |
| PL | Parietal Lobe |
| Post. SFS | Posterior Superior Frontal Sulcus |
| ReG | Rectal Gyrus |
| SFG | Superior Frontal Gyrus |
| SFS | Superior Frontal Sulcus |
| SMG | Supramarginal Gyrus |
| spACC | subgenual Anterior Cingulate Cortex |
| SPL | Superior Parietal Lobule |
