# Supplementary material for: Complementing Engel Classification: Postoperative Scalp EEG as an Objective Indicator in SEEG‐Guided Radiofrequency Thermocoagulation—A Seizure‐Onset Pattern‐Based Stratified Analysis—
Source: Ann Clin Transl Neurol. 2026 Oct 10:10.1002/acn3.70553. Online ahead of print. doi: 10.1002/acn3.70553 (PMC13653232; doi:10.1002/acn3.70553)
Supplement: Supplementary file 2 [file ACN3-9999-0-s002.docx]

**Supplementary Figure 1. Postoperative MRI Images of Some Patients.**

Patient 3 RF target：R. SPL; R. Mid. PoCG.

Patient 5 RF target：L. Ant.-Mid. CG; L. Ant. SFG.

Patient 6 RF target：L. SFS (Ant. to PreCS).

Patient 7 RF target：R. OL

Patient 8 RF target：R. Mid. PreCG

Patient 9 RF target：R. SFG; R. Post. SFS.

Patient 14 RF target：R. Inf. PoCG; R. IPL.

Patient 16 RF target：R. PL; R. SMG.

Patient 17 RF target：L. aCG (Genu)

Patient 20 RF target：L. OL; L. PCun.

Patient 21 RF target：L. PHG; L. Amyg
